# Development of Mucosal Immunity in Children: A Rationale for Sublingual Immunotherapy?

**DOI:** 10.1155/2012/492761

**Published:** 2011-10-27

**Authors:** Aleksandra Szczawinska-Poplonyk

**Affiliations:** Department of Pediatric Pneumonology, Allergology and Clinical Immunology, Poznan University of Medical Sciences, Szpitalna Street 27/33, 60-572 Poznan, Poland

## Abstract

The mucosal immune system has bidirectional tasks to mount an effective defense against invading harmful pathogens and to suppress the immune response to alimentary antigens and commensal bacterial flora. Oral tolerance is a suppression of the mucosal immune pathway related to a specific immunophenotype of the dendritic cells and an induction of the regulatory T cells as well as with the silencing of the effector T cell response by anergy and deletion. The physiological dynamic process of the anatomical and functional maturation of the immune system occurring in children during pre- and postnatal periods is a significant factor, having an impact on the fine balance between the activation and the suppression of the immune response. In this paper, mechanisms of mucosal immunity and tolerance induction in terms of maturational issues are discussed with a special emphasis on the implications for a novel therapeutic intervention in allergic diseases via the sublingual route.

## 1. Introduction

The mucosal immune system comprises the lymphoid-associated structures of the nasal, bronchial, gastrointestinal, and genitourinary tracts as well as lacrimal, salivary, and lactating mammary glands and the synovium of joints. It is composed of a dynamic network of highly specialized components of the innate and adaptive immune responses, which give rise to the functional common mucosal immune system (CMIS) and ensure fine, organ-specific balance between activation and suppression. The fundamental challenge of mucosal immune response is to prevent effectively the entry of invading pathogens and the development and the disseminating of infection, whereas simultaneously its exposition to the external environment and to a high antigenic load elicits immune tolerance. These interrelated processes of active promotion and suppression of immunity provide a defense against microorganisms and neoplasms and protect against inflammatory pathologies such as allergy and autoimmunity as well. To maintain the immune homeostasis in the oral mucosa which represents the entry port to the gastrointestinal tract, protolerogenic mechanisms take place in this tissue and dominate over active immune responses.

The development of mucosal immunity in children is a time-dependent process initiated in the intrauterine growth and is continuous during the postnatal period. Despite the anatomical and functional immaturity of the mucosal immune system and crosstalk between innate and adaptive immune responses, infants and young children are capable of mounting effective immune defense mechanisms. However, during this age, an imperfect regulatory immune response, which is of crucial importance in developing oral mucosal immunity, may pose an increased risk of food allergies. If developing new strategies of immunotherapy which exploit the establishing of an oral mucosal tolerance has a rationale in pediatric patients is here the subject of discussion.

## 2. Mucosal Defense Mechanisms

### 2.1. Mucosal Barrier

Extensive noncellular physical barriers and chemical processes as well as cellular components constitute mucosal barriers to antigen entry in the mucosa-associated lymphoid tissue (MALT). Structural differentiation of the mucosal epithelium and the appearance of intercellular tight junctions lead to the formation of an anatomical basis for an epithelial barrier. A significant protective barrier is constituted by the presence of digestive enzymes starting in the mouth and extending down to the stomach, the small bowel, and the colon, which not only allow the process of digestion, but also modify potentially immunogenic antigens and alter antigen exposure. Mucin glycoproteins, lining the surface epithelium, produce a barrier in which particles and pathogens are trapped and protect the underlying epithelium (the so-called nonimmune exclusion) as well as serving as a reservoir for the secretory IgA [1]. A number of antimicrobial components of saliva contribute to protection against microbial colonization and infection. These include peptides such as salivary peroxidase, lysozyme, lactoferrin, cystatins, SLPI (secretory leukocyte protease inhibitor), agglutinin, peptides of the histatin family, and cathelicidin (LL-37) as well as *α*- and *β*-defensins, which are expressed and secreted by salivary glands and/or ducts. In addition to exerting an antimicrobial response, these peptides facilitate and amplify innate and adaptive immune responses [2, 3]. Interestingly, it has been recently demonstrated that the expression and antimicrobial activity of cathelicidin in the oral mucosa is induced by vitamin D [4, 5].

### 2.2. Innate Mucosal Immune Response

The crucial elements of the innate arm of immunity are pattern recognition receptors (PRRr), such as Toll-like receptors (TLRr), retinoid acid-inducible gene-I- (RIG-I-) like receptors (RLRr) and nucleotide-binding oligomerization domain (NOD)-like receptors (NLRr), which recognize pathogen-associated molecular patterns (PAMPs) and molecular structures specific for microbial pathogens. Signaling by pattern-recognition receptors on antigen-presenting cells induces costimulatory molecules and cytokines, and furthermore activating a response in B and T cells. The stimulation of Toll-like receptors by PAMPs initiates signaling cascades that involve a number of proteins, including MyD88 (myeloid differentiation primary response gene 88), IRAK (interleukin- (IL-) receptor associated kinase), Toll/IL-1 receptor (TIR) domain-containing adapter-inducing interferon (IFN)-*β* (TRIF). Subsequent activation of nuclear factor NF*κ*B triggers the production of proinflammatory cytokines, such as tumor necrosis factor (TNF)-*α*, IL-1, and IL-12, which direct adaptive immune responses. Functional cooperation and cross-regulation between TLRs and the complement components, such as C1q, properdin, and the mannose-binding lectin, using the pattern recognition strategy has been demonstrated. The complement-TLR interplay reinforces innate immunity or regulates excessive inflammation, through synergistic or antagonistic interactions [6]. Moreover, ficolin molecules (L-, M-, and H-ficolin) which recognize pathogen-associated molecular patterns and initiate the lectin pathway of complement activation are thus a further component of mucosal immunity linking innate and adaptive immune responses [7].

The migration of T and B cells from the lymph nodes to the mucosa, which is related to the activation, recirculation, and homing of lymphocytes is controlled by the specific system of integrin-type molecules, selectins [8] and chemokines [9].

### 2.3. Adaptive Immune Response

The mucosal immune system has generated two arms of an adaptive response, namely, antigen exclusion, performed by different T cell subsets, B cells and secretory antibodies to inhibit or modulate adherence or colonization of microorganisms and prevent penetration of potentially harmful antigens, as well as suppressive mechanisms to avoid overreaction against innocuous substances which are in contact with the mucosal surfaces. A central role in this interrelated network of lymph cell subsets is played by dendritic cells (DCs), which are important initiators of adaptive immunity. DC prime naïve T cells to expand clonally and differentiate into T-cell subsets—T helper Th1, Th2, Th17, or T regulatory (Treg) cells. It has been demonstrated that these cells may have discrete subsets and functions, namely, CXC3CR1(+)DC which promote Th1/Th17 cell differentiation, whereas CD103(+)DC induce Treg cell differentiation on an animal model [10]. At the mucosal site, dendritic cells and T lymph cells interact with B cells promoting their differentiation and the production of antibodies. Most immunoglobulin class-switching is T cell dependent; however, it has been demonstrated that T-cell independent process may also occur, whereby DC and mucosal epithelial cells excrete BAFF (B cell activating factor belonging to the TNF family) or APRIL (a proliferation inducing ligand) directly stimulating B cells to become IgA secreting plasma cells [11, 12]. IgA is the major class of antibodies in mucosal secretions and occurs predominantly in a secretory IgA (sIgA) form along with secretory IgM (sIgM). The distribution of IgA subclasses varies at different mucosal sites—in the salivary glands and oral mucosa IgA1, associated with a response to protein antigens predominates, whereas in the distal portion of the gastrointestinal tract mainly IgA2, active in response to polysaccharide antigens is found [13]. The recently characterized Th17 lymphocytes subset is important for the induction of a mucosal adaptive immune response. It has been demonstrated that IL-17 elevates secretory IgA levels by upregulating A polymeric immunoglobulin receptor expression in mucosal epithelia [14] and promotes B cell differentiation in IgA-secreting plasma cells on a T cell-independent manner [15]. Furthermore, IL-17 plays a protective role in infectious diseases at the oral mucosa through the recruitment of neutrophils and extracellular pathogen clearance [16].

## 3. Maturation of Mucosal Immunity in Children

### 3.1. Ontogeny of Mucosal Immunity During Prenatal Period

The structures of the mucosal immune system are fully developed by the 28 gestational week, and thus, premature infants older than 28 weeks of gestation are capable of mounting an effective mucosal immune response [13]. Mucosal epithelial barrier formation commences from gestational week 10; however, the immaturity of intercellular tight junctions results in paracellular permeability, which is advantageous in the intrauterine period by allowing a bidirectional exchange of bioactive molecules between amniotic fluid and fetal serum [17]. Salivary amylase, lysozyme, and lactoferrin concentrations are most prominent in the fetal period as demonstrated by Thrane et al. [18], affording nonspecific protection in the absence of effective specific secretory immunity. Indeed, in the absence of intrauterine infection, the mucosal immune system is essentially devoid of IgA-containing lymphocytes, and until birth, there are no active B cells in the intestinal lymphoid follicles or bronchus-associated lymphoid tissue (BALT). In the salivary glands, IgM positive cells have been reported from 110–140 days of gestation and IgA positive cells with predominance of IgA1 subclass at 180 days of gestation, but no IgD-, IgG-, and IgE-producing cells have been identified by the same authors [18]. The appearance of secretory antibodies in utero can be explained by the possibility that a fetus could have been exposed to bacterial or viral protein antigens or by the induction of a fetal immune response by maternal anti-idiotypic antibodies.

### 3.2. Postnatal Maturation of Mucosal Immunity

Mucosal permeability is rapidly reduced within the first 48 hours after birth. In the oral mucosa, disappearance of maternally derived IgG reflects this postnatal mucous membrane closure [19]. This maturational process of the gut barrier function is enhanced by human milk [20] as well as by early intestinal colonization with lactobacilli and bifidobacteria [21]. The rapid increase of innate defense factors, such as salivary lysozyme, lactoferrin, and amylase during the first six postnatal months reported by Thrane et al. [18] may provide the infant necessary protection during the period when specific adaptive immunity at mucosal sites is not fully developed. Postnatal maturation of B lymph cell at mucosal surfaces has its peak from birth until the 12 week of age and corresponds with the increase of IgG-producing cells in the parotid salivary glands. Secretory IgM antibodies appear in mucosal secretions only transiently during early infancy. IgA-producing immunocytes, albeit they increase in number during neonatal period and reach an initial peak about 4–6 postnatal weeks, approach the low normal adult level at about 18 months of age, subsequently with small increase throughout early childhood [17]. Qualitative changes in secretory IgA are also seen after birth when a switch from monomeric to polymeric sIgA is observed, indicating maturation of the mucosal secretory immune system. Furthermore, in the perinatal period, IgA1subclass, associated with responses to protein antigens, predominates in mucosal secretions, but IgA2 subclass increases rapidly after birth by 6 months of age to approach adult proportions. This pattern may also reflect postnatal changes in the type and load of antigenic exposure, in particular to polysaccharide antigens [22]. Interestingly, in preterm infants sIgA appears in secretions at a similar chronological age as in full-term infants although its concentrations may be significantly lower until the eighth month of life, as reported by Kuitonen and Savilahti [23]. However, in contrast to these data, Seidel et al. [24] demonstrated comparable salivary IgA levels in preterm and full-term infants, suggesting that the development of the oral mucosal immunocompetence in preterm infants is well established within the first 9 months of life. In preschool children, the developmental profile of mucosal immunity depends on the degree of antigenic challenge they experience as well as on the exposure to hazardous environmental agents, such as tobacco smoke [25].

## 4. The Phenomenon of Mucosal Tolerance

### 4.1. Induction of Tolerance

In parallel to local defense mechanisms which protect against invading pathogens, the mucosal immune system has developed specialized regulatory and anti-inflammatory mechanisms for eliminating or tolerating harmless food and airborne antigens as well as commensal microorganisms. Mucosal tolerance induction is, therefore, an active process and is seen as preferential the Th2 skewed immune response and the downregulation of Th1 cell-mediated delayed type hypersensitivity and antibody production. These complex regulatory mechanisms include clonal deletion of T cells, clonal anergy, antigen-driven immunosuppression as well as active inhibition by coinhibitory receptors [26]. Many different CD4+ T regulatory (Treg) cell subsets have been identified capable of inhibiting the responses of effector T cells. Thymus-derived CD4+CD25+ Foxp3 (forkhead box protein 3)+Treg cells play a fundamental role in maintaining self-tolerance and preventing autoimmunization as well as contributing to tolerance of nonself antigens by the inhibition of immune responses directed at commensal bacteria in the intestine [27]. Mucosal Foxp3+ cells have been identified in the small and large intestinal mucosa as early as 23 weeks of gestational age, indicating a potential for intestinal immune regulation immediately after birth [28]. In contrast to thymus-derived Treg cells, adaptive Treg cells, which are peripherally induced after feeding protein, are essential for mucosal tolerance. These include TGF-*β*- (transforming growth factor *β*-) producing Th3 cells, type 1 T regulatory cells (Tr1) which produce IL-10 as well as Foxp3+Treg cells. The active suppressive mechanisms may also induce a “bystander effect” in that suppressive cytokines released by regulatory T cells in an antigen-specific pattern may also suppress ongoing immune response to an unrelated but anatomically colocalized antigen [29]. 

It is worth of note that the term “mucosal tolerance” is widely used to describe tolerance induction occurring in the intestinal MALT (mucosa-associated lymphoid tissue), represented by B cell follicles and M cell containing lymphoid epithelium, where the uptaken antigens are passed to APC (antigen-presenting cells), such as dendritic cells, macrophages, and B cells. However, in contrast to the intestine, the oral mucosa lacks inductive site represented by MALT and most likely local organized lymphoid tissue and regional lymph nodes play a role in the induction of oral mucosal tolerance [26]. 

**Figure 1:**
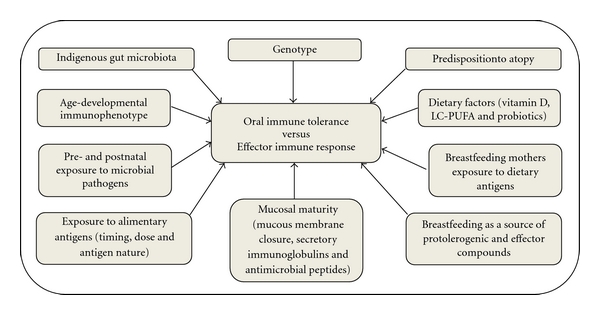
Exo- and endogenous biological factors determining mucosal immune response profile in childhood.

Dendritic cells, the most important components orchestrating the mucosal tolerance in the gastrointestinal tract, have an intrinsic noninflammatory activation state and a rich repertoire of receptors expressed by these cells, such as high-affinity receptor for IgE (Fc*ε*RI), high- and low-affinity receptors for IgG (Fc*γ*RI and Fc*γ*RII, resp.), Toll-like receptors (TLR)2, and TLR4 and LPS (lipopolysaccharide) receptor CD14, are of crucial importance in the induction of antigen-specific regulatory T cells. Furthermore, several factors, such as the nature and dose of antigen, the frequency of its administration, age at first antigen exposure, maternal dietary exposure during pregnancy and breastfeeding, antigen transmission via breast milk, as well as genetic background and immunological status of the child influence the fine balance between tolerance and effector response [29]. Exo- and endogenous biological factors determining mucosal immune response profile in childhood are summarized in Figure 1.

### 4.2. Role of Breastfeeding

The newborn and infant gut is hypersensitive to proinflammatory stimuli and vulnerable to pathogens. Breastfeeding not only favors the transmission of immunocompetence from the mother to the infant, as reviewed by Chirico et al. [30], but also has immunomodulatory and anti-inflammatory properties. The dietary antigens present in breast milk coupled with immunosuppressive cytokines, such as IL-10 and TGF*β*, promote tolerance to food antigens and gut microflora. It has been demonstrated in the study by Field et al. [31] that long chain polyunsaturated fatty acids in human milk alter the infant's ability to produce cytokines enhance the anti-inflammatory effect of IL-10. Soluble TNF-*α* (tumor necrosis factor) receptors and IL-1RA (interleukin 1 receptor antagonist) in human milk effectively inhibit inflammatory response elicited by TNF-*α* and IL-1, respectively [32], and IL-10 exhibits a suppressive effect on IL-8 and neutrophilic inflammation [33]. Human milk also contains hormones, such as epidermal growth factor (EGF), insulin-like growth factor (IGF), as well as adiponectin, which modulate the immune system by the regulation of cytokine expression [20].

## 5. Mucosal Tolerance: An Implication for Sublingual Immunotherapy

### 5.1. Oral Mucosal Microenvironment

In the oral mucosa the network of resident dendritic cells (DCs) is mainly composed of the myeloid DC from the Langerhans cell (LC) subtype, expressing CD1a and CD207 antigens (HTA1 and langerin, the LC specific lectin, corresponding with the mannose-containing oligosaccharide receptor, respectively), costimulatory molecules, such as B7.1 (CD80) and B7.2 (CD86) as well as other myeloid markers, eg CD11b (a complement components receptor). These cells are also equipped with a very specific receptor repertoire, such as a high-affinity receptor for IgE (Fc*ε*RI) resulting in allergen uptake and IgE binding to specific receptors on their surfaces. Interestingly, cross-linking of Fc*ε*RI on dendritic cells results in the induction of both pro- and, most importantly, anti-inflammatory mediators, such as IL-10 [34] and indoleamine 2,3-dioxygenase (IDO) [35], which is involved in the suppression of T cell responses and tolerance. The expression of high- and low-affinity receptors for IgG containing an immunoreceptor tyrosine inhibitory motif (ITIM) enhances the induction of antigen-specific regulatory T cells, as shown by Samsom et al. on an animal model [36]. Furthermore, TLR4 ligation on the oral DC surface leads to a subsequent induction of Foxp3 expressing as well as IL10 and TGF*β* producing regulatory T cells [37], which are key players in oral mucosal tolerance. These unique properties of DC to drive Treg cells differentiation relate to their being conditioned by commensal bacteria, TGF*β* and IL-10, their expression of *α*_E_ *β*_7_ integrin (CD103) and retinoid acid [38].

### 5.2. Immunological Mechanisms of Sublingual Immunotherapy (SLIT)

Multidirectional tolerogenic properties of an oral immune response warrant antigen-specific tolerance induction. Dendritic cells in the oral mucosa, which exhibit the high affinity receptor for IgE Fc fragment, take up allergens administered in SLIT and induce specific immune responses. An increase of serum IgG4 and IgA, noninflammatory and noncomplement binding isotypes as well as reduced allergen specific IgE locally in the target organ have been noted in the occurrence of increased TGF-*β* and IL-10 in allergen specific peripheral blood mononuclear cells [39]. Similarly, in the clinical study comprising a group of asthmatic/rhinitis pediatric patients, reported by Eifan et al. [40] the immunological mechanisms of SLIT were associated with significant increases of TGF-*β* and IL-10. Furthermore, T regulatory cell function has also been demonstrated by O'Hehir et al. [41], leading to the suppression of the allergen specific effector T (CD4+CD25-CD127hi) cell proliferation and cytokine production. In a recent study of Angelini et al. [42] the downregulation of the costimulatory molecule CD86 on blood dendritic cells, increased IL-10 and decreased IL-12 production have been demonstrated in a group of ten children with allergic asthma and house dust mites sensitivity after 12 month of SLIT. These significant functional alterations of dendritic cells may contribute to decreased T cell activation and a shift toward regulatory activity.

### 5.3. Efficacy and Safety of SLIT in Children

In the light of the aforementioned considerations regarding developmental issues of mucosal tolerance in children as well as multiple endo- and exogenous factors which may have an important impact on its outcome, important questions arise with regard to the efficiency and safety of SLIT. In meta-analysis studies performed by several investigators [43–45], comprising of pediatric patients with allergic asthma treated with SLIT, a significant reduction in the symptom scores and the use of rescue medication as well as an improvement in lung function have been demonstrated. It has been well established that SLIT requires a high allergen dose for its efficiency to facilitate a take-up of sufficient amounts of allergens by sentinel dendritic cells within the oral mucosa or due to a lack of adjuvants by sublingual administration [46]. Even though a high dose and long courses of medication are necessary, SLIT is a safe therapeutic option for children, as has been recently reported by Ferrés et al. [47]; although, in this study, the mild and local adverse reaction rate was at 23%; however, none of the cases from the study group showed an anaphylactic reaction. Similarly, in the clinical study by Eifan et al. [40] it was demonstrated that SLIT was associated with clinical improvement and proved to be a safe mode of immunotherapy. Therefore, as has been stated by Wahn [48], it seems likely that the induction of tolerance via the sublingual route to prevent the outset of allergic asthma, even in younger children, will soon be addressed in clinical studies.

## 6. Concluding Remarks

Mucosal immunity is characterized by a specific maturational pattern initiated in the intrauterine fetal development and continued during the neonatal period, and in infancy and childhood, dynamically leading to a highly specialized immune response. At mucosal sites, a subtle balance occurs between effective defense mechanisms against the invasion of harmful pathogens and triggers the limitation of effector immune reactions to food antigens and commensal flora. Important factors, such as genetic predisposition and the age of the host, pre- and postnatal exposure to antigens, as well as the properties and the dose of antigen contribute to the development of mucosal tolerance, this being the rationale of critical importance for sublingual immunotherapy. The results of hitherto prevailing clinical studies suggest the efficacy and safety of this treatment option in children, hereby opening new perspectives in pediatric allergology.

## References

[1] Mayer L (2003). Mucosal immunity. *Pediatrics*.

[2] Dale BA, Fredericks LP (2005). Antimicrobial peptides in the oral environment: expression and function in health and disease. *Current Issues in Molecular Biology*.

[3] Gomes PDS, Fernandes MH (2010). Defensins in the oral cavity: distribution and biological role. *Journal of Oral Pathology and Medicine*.

[4] McMahon L, Schwartz K, Yilmaz O, Brown E, Ryan LK, Diamond G (2011). Vitamin D-mediated induction of innate immunity in gingival epithelial cells. *Infection and Immunity*.

[5] Gombart AF (2009). The vitamin D-antimicrobial peptide pathway and its role in protection against infection. *Future Microbiology*.

[6] Hajishengallis G, Lambris JD (2010). Crosstalk pathways between Toll-like receptors and the complement system. *Trends in Immunology*.

[7] Holmskov U, Thiel S, Jensenius JC (2003). Collectins and ficolins: humoral lectins of the innate immune defense. *Annual Review of Immunology*.

[8] Dwivedy A, Aich P (2011). Importance of innate mucosal immunity and the promises it holds. *International Journal of General Medicine*.

[9] Ito T, Carson WF, Cavassani KA, Connett JM, Kunkel SL (2011). CCR6 as a mediator of immunity in the lung and gut. *Experimental Cell Research*.

[10] Niess JH, Adler G (2010). Enteric flora expands gut lamina propria CX_3_CR1^+^ dendritic cells supporting inflammatory immune responses under normal and inflammatory conditions. *Journal of Immunology*.

[11] Puga I, Cols M, Cerutti A (2010). Innate signals in mucosal immunoglobulin class switching. *Journal of Allergy and Clinical Immunology*.

[12] Chorny A, Puga I, Cerutti A (2010). Innate signaling networks in mucosal IgA class switching. *Advances in Immunology*.

[13] Gleeson M, Cripps AW (2004). Development of mucosal immunity in the first year of life and relationship to sudden infant death syndrome. *FEMS Immunology and Medical Microbiology*.

[14] Jaffar Z, Ferrini ME, Herritt LA, Roberts K (2009). Cutting edge: lung mucosal Th17-mediated responses induce polymeric Ig receptor expression by the airway epithelium and elevate secretory IgA levels. *Journal of Immunology*.

[15] Doreau A, Belot A, Bastid J (2009). Interleukin 17 acts in synergy with B cell-activating factor to influence B cell biology and the pathophysiology of systemic lupus erythematosus. *Nature Immunology*.

[16] Guglani L, Khader SA (2010). Th17 cytokines in mucosal immunity and inflammation. *Current Opinion in HIV and AIDS*.

[17] Maheshwari A, Zemlin M (2006). Ontogeny of the intestinal immune system. *Immunology and Infection*.

[18] Thrane PS, Rognum TO, Brandtzaeg P (1991). Ontogenesis of the secretory immune system and innate defence factors in human parotid glands. *Clinical and Experimental Immunology*.

[19] Gleeson M, Cripps AW, Clancy RL, Husband AJ, Hensley M,J, Leeder SR (1982). Ontogeny of the secretory immune system in man. *Australian and New Zealand Journal of Medicine*.

[20] Newburg DS, Walker WA (2007). Protection of the neonate by the innate immune system of developing gut and of human milk. *Pediatric Research*.

[21] Sherman MP, Bennett SH, Hwang FF, Yu C (2004). Neonatal small bowel epithelia: enhancing anti-bacterial defense with lactoferrin and Lactobacillus GG. *BioMetals*.

[22] Weemaes C, Klasen I, Göertz J, Beldhuis-Valkis M, Olafsson O, Haraldsson A (2003). Development of immunoglobulin a in infancy and childhood. *Scandinavian Journal of Immunology*.

[23] Kuitonen M, Savilahti E (1995). Mucosal IgA, mucosal cow’s milk antibodies, serum cow’s milk antibodies and gastrointestinal permeability in infants. *Pediatric Allergy and Immunology*.

[24] Seidel BM, Schulze B, Schubert S, Borte M (2000). Oral mucosal immunocompetence in preterm infants in the first 9 months of life. *European Journal of Pediatrics*.

[25] Ewing P, Otczyk DC, Occhipinti S, Kyd JM, Gleeson M, Cripps A (2010). Developmental profiles of mucosal immunity in pre-school children. *Clinical and Developmental Immunology*.

[26] Novak N, Haberstok J, Bieber T, Allam JP (2008). The immune privilege of the oral mucosa. *Trends in Molecular Medicine*.

[27] du Pre MF, Samsom JN (2011). Adaptive T-cell responses regulating oral tolerance to protein antigen. *Allergy*.

[28] Weitkamp JH, Rudzinski E, Koyama T (2009). Ontogeny of FOXP3^+^ regulatory T cells in the postnatal human small intestinal and large intestinal lamina propria. *Pediatric and Developmental Pathology*.

[29] Strobel S (2001). Immunity induced after a feed of antigen during early life: oral tolerance v. sensitisation. *Proceedings of the Nutrition Society*.

[30] Chirico G, Marzollo R, Cortinovis S, Fonte C, Gasparoni A (2008). Antiinfective properties of human milk. *Journal of Nutrition*.

[31] Field CJ, Thomson CA, Van Aerde JE (2000). Lower proportion of CD45R0^+^ cells and deficient interleukin-10 production by formula-fed infants, compared with human-fed, is corrected with supplementation of long-chain polyunsaturated fatty acids. *Journal of Pediatric Gastroenterology and Nutrition*.

[32] Buescher ES (2001). Anti-inflammatory characteristics of human milk: how, where, why. *Advances in Experimental Medicine and Biology*.

[33] Field CJ (2005). The immunological components of human milk and their effect on immune development in infants. *Journal of Nutrition*.

[34] Novak N, Bieber T, Katoh N (2001). Engagement of Fc*ε*RI on human monocytes induces the production of IL-10 and prevents their differentiation in dendritic cells. *Journal of Immunology*.

[35] von Bubnoff D, Matz H, Frahnert C (2002). Fc*ε*RI induces the tryptophan degradation pathway involved in regulating T cell responses. *Journal of Immunology*.

[36] Samsom JN, van Berkel LA, Van Helvoort JM (2005). Fc*γ*RIIB regulates nasal and oral tolerance: a role for dendritic cells. *Journal of Immunology*.

[37] Allam JP, Peng WM, Appel T (2008). Toll-like receptor 4 ligation enforces tolerogenic properties of oral mucosal Langerhans cells. *Journal of Allergy and Clinical Immunology*.

[38] Weiner HL, da Cunha AP, Quintana F, Wu H (2011). Oral tolerance. *Immunological Reviews*.

[39] Akdis CA, Barlan IB, Bahceciler N, Akdis M (2006). Immunological mechanisms of sublingual immunotherapy. *Allergy*.

[40] Eifan AO, Akkoc T, Yildiz A (2010). Clinical efficacy and immunological mechanisms of sublingual and subcutaneous immunotherapy in asthmatic/rhinitis children sensitized to house dust mite: an open randomized controlled trial. *Clinical and Experimental Allergy*.

[41] O’Hehir RE, Gardner LM, de Leon MP (2009). House dust mite sublingual immunotherapy: the role for transforming growth factor-*β* and functional regulatory T cells. *American Journal of Respiratory and Critical Care Medicine*.

[42] Angelini F, Pacciani V, Corrente S (2011). Dendritic cells modifi cation during sublingual immunotherapy in children with allergic symptoms to house dust mites. *World Journal of Pediatrics*.

[43] Ozdemir C, Yazi D, Gocmen I (2007). Efficacy of long-term sublingual immunotherapy as an adjunct to pharmacotherapy in house dust mite-allergic children with asthma. *Pediatric Allergy and Immunology*.

[44] Penagos M, Passalacqua G, Compalati E (2008). Metaanalysis of the efficacy of sublingual immunotherapy in the treatment of allergic asthma in pediatric patients, 3 to 18 years of age. *Chest*.

[45] Marseglia GL, Incorvaia C, La Rosa M, Frati F, Marcucci F (2009). Sublingual immunotherapy in children: facts and needs. *Italian Journal of Pediatrics*.

[46] Moingeon P, Batard T, Fadel R, Frati F, Sieber J, Van Overtvelt L (2006). Immune mechanisms of allergen-specific sublingual immunotherapy. *Allergy*.

[47] Ferrés J, Justicia JL, García MP, Muñoz-Tudurí M, Alvà V (2011). Efficacy of high-dose sublingual immunotherapy in children allergic to house dust mites in real-life clinical practice. *Allergologia et Immunopathologia*.

[48] Wahn U (2010). Sublingual immunotherapy in children—ready for prime time?. *Pediatric Allergy and Immunology*.

